# Concussion Among Children in the United States General Population: Incidence and Risk Factors

**DOI:** 10.3389/fneur.2021.773927

**Published:** 2021-11-01

**Authors:** Nathan E. Cook, Grant L. Iverson

**Affiliations:** ^1^Department of Physical Medicine and Rehabilitation, Harvard Medical School, Boston, MA, United States; ^2^MassGeneral Hospital for Children Sports Concussion Program, Boston, MA, United States; ^3^Spaulding Rehabilitation Hospital, Charlestown, MA, United States; ^4^Spaulding Research Institute, Charlestown, MA, United States

**Keywords:** mild traumatic brain injury, head trauma, pediatric, traumatic injury, epidemiology

## Abstract

The objective of this study was to examine the incidence of concussion and risk factors for sustaining concussion among children from the United States general population. This prospective cohort study used data from the Adolescent Brain Cognitive Development (ABCD) Study^®^. Children were recruited from schools across the US, sampled to reflect the sociodemographic variation of the US population. The current sample includes 11,013 children aged 9 to 10 years old (47.6% girls; 65.5% White) who were prospectively followed for an average of 1 year (mean = 367.9 days, SD = 40.8, range 249–601). The primary outcome was caregiver-reported concussion during a 1 year follow-up period. Logistic regression was used to determine which potential clinical, health history, and behavioral characteristics (assessed at baseline) were prospectively associated with concussion. In the 1 year follow-up period between ages 10 and 11, 1 in 100 children (*n* = 123, 1.1%) sustained a concussion. In univariate models, three baseline predictors (ADHD, prior concussion, and accident proneness) were significantly associated with sustaining a concussion. In a multivariate model, controlling for all other predictors, only prior concussion remained significantly associated with the occurrence of a concussion during the observation period (Odds Ratio = 5.49, 95% CI: 3.40–8.87). The most robust and only independent prospective predictor of sustaining a concussion was history of a prior concussion. History of concussion is associated with 5.5 times greater odds of sustaining concussion between ages 10 and 11 among children from the general US population.

## Introduction

Concussion among children is a source of significant concern among parents, school personnel, healthcare providers, and policy makers (1–7). It is of particular clinical and public policy importance to better understand risk factors for sustaining concussion. A few potential risk factors for pediatric sport-related concussion have been identified. Systematic reviews have identified strong evidence that history of a previous concussion is a risk factor for sustaining a future concussion (8, 9), with previously concussed youth athletes having more than 3 times greater risk of sustaining a future concussion (pooled risk ratio = 3.64; 95% CI: 2.68–4.96) (8). In addition, several retrospective studies of youth athletes report that boys have a greater lifetime history of concussion than girls (10–12). However, evidence regarding other potential risk factors, including gender, as well as aggressive athlete behavior, and body weight/body mass, have been inconsistent or have been classified as having low levels of certainty (9). Moreover, available literature suggests that having attention-deficit/hyperactivity disorder (ADHD) may be a risk factor for sustaining concussion. When asked to report their health history in the context of sports participation, children, adolescents, and young adults with ADHD self-report greater lifetime rates of concussion than those who do not have ADHD (11–16). Further, a recent case-control study using the Adolescent Brain Cognitive Development (ABCD) Study^®^ database reported that children with ADHD were significantly more likely to have a lifetime history of concussion compared to children who did not have ADHD (17). However, the literature on ADHD as a risk factor for sustaining concussion is methodologically limited in important ways, such as being cross-sectional, retrospective, and subject to recall bias.

We found no prior prospective studies relating to risk factors for sustaining concussion among children in the general population. Using data from the ABCD Study^®^, the largest long-term study of brain development and child health in the United States (18, 19), we examined potential risk factors, assessed when children were 9 or 10 years old, and the occurrence of concussion during a 1 year follow-up period in a large, diverse, representative cohort of children from the U.S. general population. We hypothesized that (i) prior concussion, (ii) ADHD, (iii) impulsive tendencies, (iv) accident proneness, (v) male gender, and (vi) greater body mass index would be associated with increased risk of sustaining concussion.

## Materials and Methods

### Participants

Participants were drawn from the most recent curated data release (3.0) from the ABCD study (18), a comprehensive longitudinal study involving 21 research sites across the United States. Data release 3.0 includes concussion interview results from the 1 year follow-up study visit for 11,194 children, of which 164 were missing data on ADHD status, 3 children were missing Child Behavior Checklist (CBCL) data, 1 child was missing gender, 4 were missing height recordings, and 2 were missing weight recordings. We excluded children with presumed data entry errors, including 2 children with height recordings of 4 inches and 2 children with weight recordings of 11 pounds. We also excluded 1 child whose follow-up interview date was just 14 days after the baseline interview and 2 children due to having sustained an injury with loss of consciousness lasting >30 min. The final sample for this study included 11,013 children. The mean age of the sample was 9.9 years (*SD* = 0.6, range: 9.0–10.9) at baseline assessment and 10.9 years (*SD* = 0.6, range: 9.7–12.4) at the 1 year follow-up. The sample was roughly equally split between girls (47.6%) and boys (52.4%). The sample racial and ethnic composition as well as socioeconomic status are summarized in Table 1. All sociodemographic information was provided by caregivers. The average time between baseline and follow-up assessment was 367.9 days (SD = 40.8, median = 365, range 249–601).

**Table 1:** Sample demographics.

	**Total**
	***N* = 11,013**
Age at baseline, *M* (*SD*)	9.9 (0.6)
Age at follow-up, *M* (*SD*)	10.9 (0.6)
**Gender**, ***n*** **(*****%*****)**	
Girls	5,244 (47.6)
Boys	5,769 (52.4)
**Race**, ***n*** **(*****%*****)**	
White	7,111 (65.5)
Black/African American	1,633 (15.0)
American Indian	55 (0.5)
Pacific Islander	13 (0.1)
Asian	299 (2.1)
Other race	455 (4.2)
Multiracial^∧^	1,361 (12.5)
**Ethnicity**, ***n*** **(*****%*****)**	
Hispanic/Latinx	2,163 (19.9)
Not Hispanic/Latinx	8,713 (80.1)
**Household Income**, ***n*** **(*****%*****)**	
Less than $35,000	1,765 (19.3)
$35,000–$99,999	3,379 (36.9)
≥$100,000	4,017 (43.8)

*M, mean; SD, standard deviation, and n, sample size. The frequencies of demographic categories do not necessarily sum to the total N for that column due to a small percentage of participants having missing data on that variable*.

∧ *Children were classified as Multiracial if caregivers indicated the child belonged to more than one racial group*.

### Measures

#### The Ohio State Traumatic Brain Injury Screen-Short Modified (OTBI)

The OTBI is a questionnaire for caregivers assessing whether their child has sustained a traumatic brain injury (20). Parents are asked the following 4 questions to determine the occurrence of a head injury:

1. Has your child ever been hospitalized or treated in an emergency room following an injury to his/her head or neck?;
2. Has your child ever injured his/her head or neck in a car accident or from crashing some other moving vehicle like a bicycle, motorcycle, or ATV?;
3. Has your child ever injured his/her head or neck in a fall or from being hit by something? (For example, falling from a bike or horse, rollerblading, falling on ice, being hit by a rock); Has your child ever injured his/her head or neck playing sports or on the playground?; and
4. Has your child ever injured his/her head or neck in a fight, from being hit by something, or from being shaken violently?

If a prior injury is indicated, the parent is asked questions to determine injury severity [i.e., (a) Was he/she knocked out or did he/she lose consciousness? If yes, how long?; and (b) Was he/she dazed or did he/she have a gap in his/her memory from the injury?]. For this study, after excluding children with a reported loss of consciousness >30 min, we examined prior concussions, defined as any positive response to the 4 injury occurrence questions with a reported loss of consciousness (lasting <30 min) and/or the child feeling dazed or having a memory gap, assessed at baseline as a predictor/covariate. Additionally, we examined caregiver responses to the OTBI re-administered at the follow-up visit, for which caregivers were asked to indicate any injuries occurring “Since we last saw you on (date of baseline assessment).” Concussions occurring between the baseline and follow-up interviews (reported at the follow-up assessment) were the primary outcome for this study. Of note, given the wording of the OTBI questions, sport-related concussions could not be isolated or separated from several other injury mechanisms, including falls and being hit by something (see question 3 above).

#### Kiddie Schedule for Affective Disorders and Schizophrenia for DSM-5 (KSADS-5)

The KSADS-5 (21) is a structured interview to assess Diagnostic and Statistical Manual of Mental Disorders-Fifth Edition, i.e., DSM-5 (22) diagnoses, and it has been translated into a computerized assessment (23). We used results from the self-administered parent version administered during the baseline assessment. Meeting formal diagnostic criteria for current ADHD at baseline was used as a predictor/covariate.

#### Child Behavior Checklist (CBCL)

The CBCL is a standardized caregiver-report questionnaire of a child's emotional and behavioral functioning (24). The caregiver responds to items by endorsing “Not True,” “Somewhat or Sometimes True,” or “Very True or Often True” based on the preceding 6 months. For this study, we examined caregiver responses on 2 CBCL items collected at the baseline assessment: item 36 “Gets hurt a lot, accident prone” and item 41 “Impulsive or acts without thinking.” Item responses were recoded as binary (0 for Not True, and 1 for either of the other responses) and included as predictors/covariates.

#### Youth Anthropometrics Modified From PhenX Toolkit

We used children's height (inches) and weight (pounds) averaged across three measurements at the time of their baseline assessment to calculate their body mass index (BMI) as weight in pounds divided by the height in inches divided by height in inches multiplied by 703 (25). BMI-for-age percentiles were referenced, and BMI was recoded into a binary variable according to the CDC (https://www.cdc.gov/healthyweight/assessing/bmi/childrens_bmi/about_childrens_bmi.html) as 1 = greater than or equal to the 85th percentile or 0 = below the 85th percentile, using the following values (85th percentile for girls: age 9 = 19, age 10 = 20; 85th percentile for boys: age 9 = 18.6, age 10 = 19.4) (25).

### Statistical Analyses

The proportion of children who sustained a concussion between the baseline and 1 year follow-up assessment was calculated. The following potential risk factors drawn from the baseline study assessment were examined: gender (girl = 1, boy = 2), prior concussion (0 = no, 1 = yes), ADHD (0 = no, 1 = yes), impulsivity (0 = no, 1 = yes), accident proneness (0 = no, 1 = yes), and BMI-for-age (0 = below the 85th percentile, 1 = greater than or equal to the 85th percentile). Logistic regressions were used to determine which potential risk factors were prospectively associated with a concussion over the 1 year follow-up period. We included each covariate in separate univariate models (unadjusted models). Next, all covariates were entered into a multivariate logistic regression in a single step (adjusted model). Bootstrapping (1,000 samples) was performed to assess risk of model over-fitting for the adjusted model. Analyses were conducted using SPSS Version 25.

## Results

In the 1 year follow-up period between ages 10 and 11, 1 in 100 children (*n* = 123, 1.1%) sustained a concussion. Unadjusted significant predictors of sustaining a concussion were ADHD [Odds Ratio (OR) = 1.72, 95% CI: 1.04–2.85], accident proneness (OR = 1.60, 95% CI: 1.04–2.48), and having sustained a prior concussion (OR = 5.99, 95% CI: 3.74–9.61). Gender (OR = 1.33, 95% CI: 0.93–1.91), impulsivity (OR = 1.22, 95% CI: 0.82–1.81), and a high body mass index (OR = 0.90, 95% CI: 0.61–1.31) were not significant independent predictors of sustaining a concussion. All six predictor variables were entered in a multivariable logistic regression, with concussion sustained during the observation period as the dichotomous outcome (see Table 2). The model was statistically significant (*p* < 0.001) and well-calibrated (Hosmer and Lemeshow, *p* = 0.75). Measures of discrimination suggested modest prediction accuracy (Nagelkerke *R*^2^ = 0.034; area under the receiver operating curve, or AUC = 0.63, *p* < 0.001; i.e., better than chance but poor for classifying individual cases). In the adjusted model, controlling for all other predictors, only prior concussion remained significantly associated with the occurrence of a concussion during the observation period (OR = 5.49, 95% CI: 3.40–8.87). All results from the adjusted bootstrapped model were consistent with results from the adjusted model.

**Table 2:** Multivariable logistic regression predicting concussion occurrence.

	**Adjusted**	**Bootstrapped**	
	**Odds ratio (CI)**	**Odds ratio (CI)**	**% Sustaining concussion^‡^**
Gender	1.26 (0.87–1.82)	1.26 (0.88–1.84)	Boys: 1.3% Girls: 1.0%
Prior concussion	5.49 (3.40–8.87)^*^	5.49 (3.33–8.78)^*^	Prior Concussion: 5.4% No Prior Concussion:1.0%
ADHD	1.41 (0.81–2.46)	1.41 (0.74–2.46)	ADHD: 1.8% No ADHD: 1.0%
Impulsivity	0.93 (0.60–1.45)	0.93 (0.55–1.47)	Impulsivity: 1.3% No Impulsivity: 1.1%
Accident proneness	1.35 (0.85–2.15)	1.35 (0.82–2.20)	Accident Prone: 1.6% Not Accident Prone: 1.0%
BMI ≥85th percentile	0.91 (0.62–1.33)	0.91 (0.58–1.33)	BMI ≥85th percentile: 1.0% BMI <85th percentile: 1.2%

*CI, 95% confidence interval; ADHD, Attention-Deficit/Hyperactivity Disorder; BMI, Body Mass Index. Bootstrapped estimates based on 1,000 samples*.

* *signifies significance at the 0.01 level*.

‡ *Parents reported an injury occurred between the time of the baseline assessment and the follow-up assessment (responded yes to at least one of the four injury occurrence questions on the OTBI) and reported either loss of consciousness or their child being dazed or experiencing a memory gap*.

## Discussion

Approximately 1 in 100 children, ages 9 and 10, sustain a concussion over the course of 1 year. Extrapolating to the United States population, based on 2019 census data ~80,000 9 or 10 year-old children will sustain a concussion each year (26). Unadjusted predictors of sustaining a concussion were ADHD, accident proneness, and having sustained a prior concussion. That is, unadjusted odds of sustaining concussion during the follow up observation period were 1.7 times greater in children with ADHD compared to those without ADHD, 1.6 times greater in children rated by their parents as being accident prone compared to those not rated as being accident prone, and nearly 6 times greater in children with a prior concussion at baseline compared to those without a prior concussion history. Gender, impulsivity, and a high body mass index were not significant independent predictors of sustaining a concussion. In a multivariate model, the only variable that was independently related to sustaining a concussion was a history of prior concussions. Adjusted odds of sustaining concussion during the follow up observation period were 5.5 times greater in children with a history of prior concussions.

The finding that a history of prior concussions is associated with increased risk for a future concussion aligns with prior studies of youth athletes (27–32). Factors underlying this association are unknown, and one could speculate that there are behavioral and environmental factors, and possibly even genetic factors, all unmeasured in this study, that could contribute. It is also theoretically possible that a prior history of concussion could result in a lowered threshold for a future concussion. It would be reasonable and plausible to assume, however, that children who sustain a concussion might have a diverse range of possible temperamental and lifestyle risk factors that could be operative.

There have been several prior studies reporting that children (11), adolescents (12, 16), and college students (14, 15) with ADHD report a greater lifetime history of concussion. Moreover, children and adolescents with ADHD are at increased risk for bodily injuries (33–36). A recent case-control study using the ABCD Study^®^ database reported that children with ADHD were significantly more likely (7.2%) to have a *lifetime* history of concussion compared to children who did not have ADHD (3.2%), and that boys and girls with ADHD did not differ in their concussion history in that study (17). However, prior studies were cross sectional, retrospective, and considered lifetime history of injury. In the present prospective study, there was a small significant association between having ADHD at ages 9 and 10 and risk for sustaining a concussion over a 1 year follow-up period in the unadjusted analyses, but not in the multivariate model. There are also several studies illustrating that male gender is associated with a greater lifetime history of concussion (11, 12, 16), including a study using the ABCD Study^®^ baseline data (37). In the present study, the lack of association with ADHD and male gender in the multivariate model might relate, in part, to the young and restricted age of the subjects and only a single year observational period. Certain risk factors might not be significant over the course of a single year, but when considering multiple years they are more likely to emerge as being predictive.

### Limitations

The occurrence of concussion was determined by caregiver report on a structured research interview and did not necessarily represent injuries diagnosed by a physician. Moreover, the OTBI injury incidence questions are broad and multifaceted (e.g., combining sports injuries with falls), thus we did not have information regarding how concussions were sustained. Ideally, we would be able to examine rates of concussion among discrete injury mechanisms. Further, the OTBI injury incidence questions are not mutually exclusive which precludes us from calculating a total number of injuries. We thus were limited to examining whether or not children had at least one concussion during the observation period. Our study includes a limited age range (i.e., children ages 9–10 monitored for 1 year and followed up at age 10–11). Also, the variables of interest relied on parent report, although we examined data collected via varying methods (semi-structured interview, questionnaires) and collected at multiple timepoints (baseline and 1 year follow-up). Future work combining various sources of information, such as medical chart linkage, will be important.

## Conclusion

In 9 and 10 year old children from the United States general population, 1 in 100 sustain a concussion over the course of a year, which translates to approximately 80,000 children of this age in the US (26). The most robust and only independent prospective predictor of sustaining a concussion in a multivariable model was history of a prior concussion. Children with a prior history of concussion had 5.5 times greater odds of sustaining a concussion, compared to children without a prior concussion history.

## Data Availability Statement

The data analyzed in this study was obtained from the National Institute of Mental Health Data Archive (NDA), the following licenses/restrictions apply: summary information on the data shared in NDA is available in the NDA Query Tool without the need for an NDA user account. To request access to record-level human subject data, you must submit a Data Access Request. Requests to access these datasets should be directed to https://nda.nih.gov/user/login_required.html?originator=%2Fuser%2Fdashboard%2Fdata_permissions.html.

## Ethics Statement

The studies involving human participants were reviewed and approved by see PMID: 29706313: Most ABCD research sites rely on a central Institutional Review Board (cIRB) at the University of California, San Diego for the ethical review and approval of the research protocol, with a few sites obtaining local IRB approval. Written informed consent to participate in this study was provided by the participants' legal guardian/next of kin.

## Author Contributions

NC helped conceptualize the study, conceptualized the statistical analyses, conducted the statistical analyses, drafted sections of the manuscript, assisted with the literature review, edited the manuscript, and approved the final manuscript. GI helped conceptualize the study, assisted with the literature review, drafted sections of the manuscript, edited the manuscript, and approved the final manuscript. Both authors approved the final manuscript as submitted and agreed to be accountable for all aspects of the work.

## Funding

NC acknowledges support from the Louis V. Gerstner III Research Scholar Award from Massachusetts General Hospital. GI acknowledges unrestricted philanthropic support from the Mooney-Reed Charitable Foundation, Heinz Family Foundation, Boston Bolts, ImPACT^®^ Applications, Inc., National Rugby League, and the Spaulding Research Institute. These entities were not involved in the study design, interpretation of data, the writing of this article or the decision to submit it for publication.

## Conflict of Interest

GI has a clinical practice in forensic neuropsychology, including expert testimony, involving individuals who have sustained mild TBIs. He has received research support from the Harvard Integrated Program to Protect and Improve the Health of NFLPA Members, and a grant from the National Football League. He serves as a scientific advisor for NanoDx™, Inc., Sway Medical, Inc., and Highmark, Inc. The remaining author declares that the research was conducted in the absence of any commercial or financial relationships that could be construed as a potential conflict of interest.

## Publisher's Note

All claims expressed in this article are solely those of the authors and do not necessarily represent those of their affiliated organizations, or those of the publisher, the editors and the reviewers. Any product that may be evaluated in this article, or claim that may be made by its manufacturer, is not guaranteed or endorsed by the publisher.
